# Mycoplasma Mucositis: A Case of Extrapulmonary Manifestation of Mycoplasma Pneumoniae

**DOI:** 10.7759/cureus.10050

**Published:** 2020-08-26

**Authors:** Travis Lambert, Mario Dervishi, Maria Markosyan Karapetyan, Zyad Iskenderian

**Affiliations:** 1 Internal Medicine, American University of the Caribbean School of Medicine, Cupecoy, SXM; 2 Internal Medicine, Ascension Providence Hospital, Southfield, USA

**Keywords:** mycoplasma pneumoniae, m. pneumoniae-induced rash, mucositis

## Abstract

We report the case of an 18-year-old male patient who presented for the evaluation of bilateral conjunctivitis, blurry vision of the left eye, penile lesions, and dysuria. The patient was admitted to the hospital due to widespread mucosal lesions and signs of disseminated infection.

Laboratory studies revealed a leukocytosis of 17.41K/µL (normal: 4K/µL - 11K/µL) with a neutrophilic predominance of 82.7%. Chlamydia trachomatis, Neisseria gonorrhoeae, human immunodeficiency virus (HIV), antinuclear antibody (ANA), hepatitis, human leukocyte antigen B27 (HLA-B27), and pathergy test for Behcet's were all negative. Mycoplasma pneumoniae IgM and IgG, herpes simplex virus-1 (HSV-1) IgG and IgM, and HSV-2 IgG were all positive.

It was determined that the cause for his lesions was likely Mycoplasma mucositis. He was treated with ceftriaxone, azithromycin, acyclovir, and methylprednisolone. After five days of treatment, complete resolution of symptoms was achieved and he was discharged home.

## Introduction

Mycoplasma pneumoniae (M. pneumoniae) is a unique bacterium that can cause a serious infection requiring hospitalizations and involve multiple organ systems [1]. The severity of extrapulmonary manifestations of M. pneumoniae are based on the host immune response and are the result of the direct invasion and/or autoimmune response [1]. Mycoplasma mucositis is a rare extrapulmonary manifestation of M. pneumoniae and presents with eruptions of rashes that affect mucous membranes with a predominance affection for the oral and ophthalmic mucosa, as well as genital and perineal regions [2-3]. Oral, ocular, and urogenital mucositis were reported in 94%, 82%, and 63% of cases, respectively [4]. Mucosal rash involvement shares similarities with erythema multiforme (EM), Stevens-Johnson syndrome (SJS), and toxic epidermal necrolysis (TEN) [5]. SJS and its more severe form TEN are characterized by keratinocyte necrosis and perivascular lymphocyte involvement which involves up to 10% of the cutaneous surface area for SJS or > 30% cutaneous involvement for TEN [6]. Steven-Johnson syndrome-toxic epidermal necrolysis overlap has characteristics of both SJS and TEN and involves 10% - 30% of the body surface area, epidermal detachment, fever, and malaise [6]. Sparing of cutaneous tissue and rash etiology accounts for the unique presentation of M. pneumoniae-associated mucositis. This pathology has been previously described as an atypical Stevens-Johnson syndrome without skin lesions. However, in the spectrum of epidermal dermatopathies, the condition is increasingly recognized as a separate entity, now termed Mycoplasma pneumoniae-associated mucositis (MPAM) [5]. Understanding nuances for each one of the rashes above becomes of significant importance due to the differences in patient management and outcomes.

## Case presentation

An 18-year-old African American male with no past medical history initially presented to an urgent care facility with bilateral conjunctivitis, blurry vision in the left eye, penile lesions, and dysuria. Three days prior to the urgent care visit, the patient began experiencing unilateral conjunctivitis. A trial of over-the-counter eye drops was attempted with no mitigation of symptoms. 

**Figure 1:**
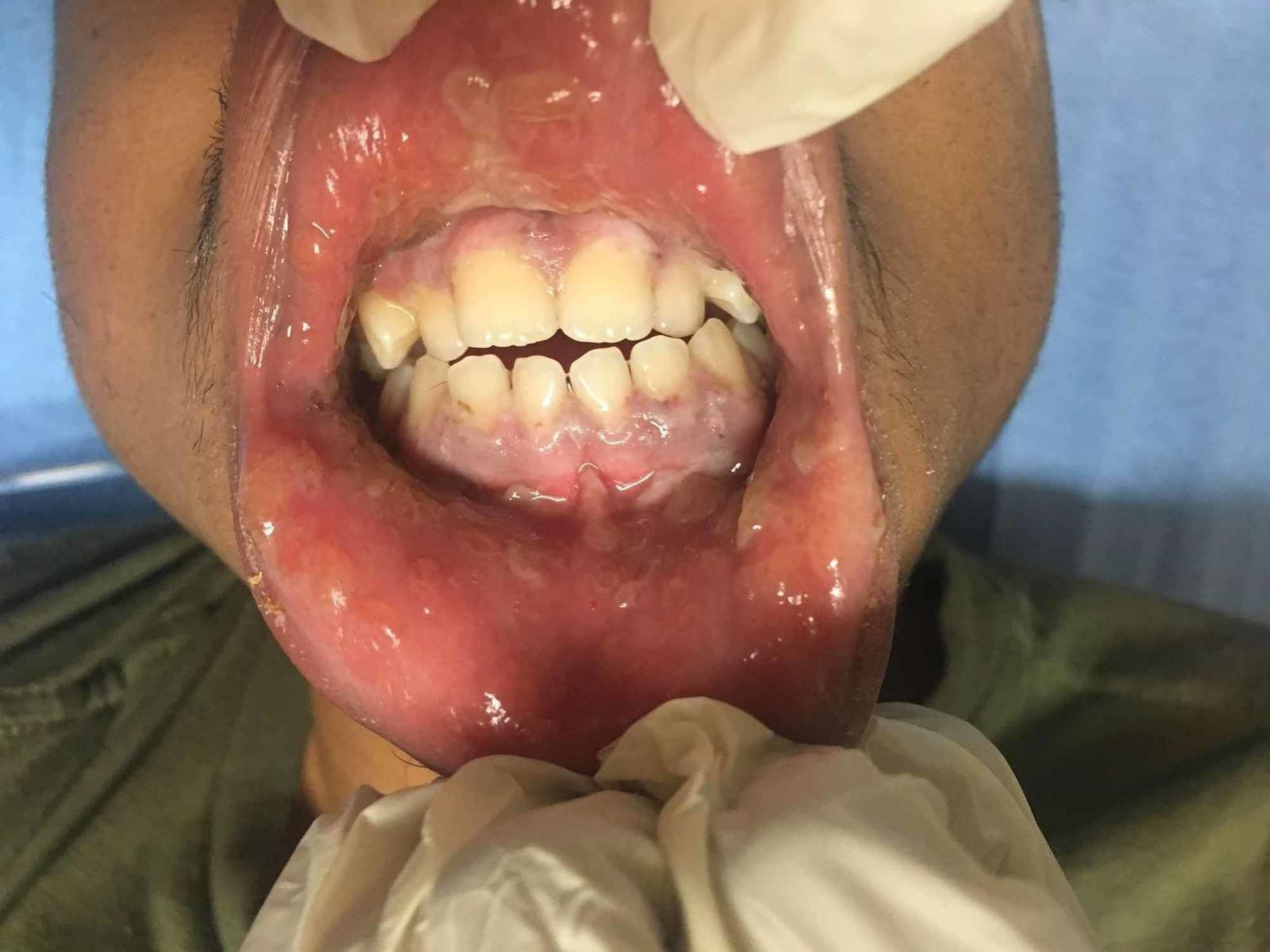
Oral sores and sloughing of the mucosa

**Figure 2:**
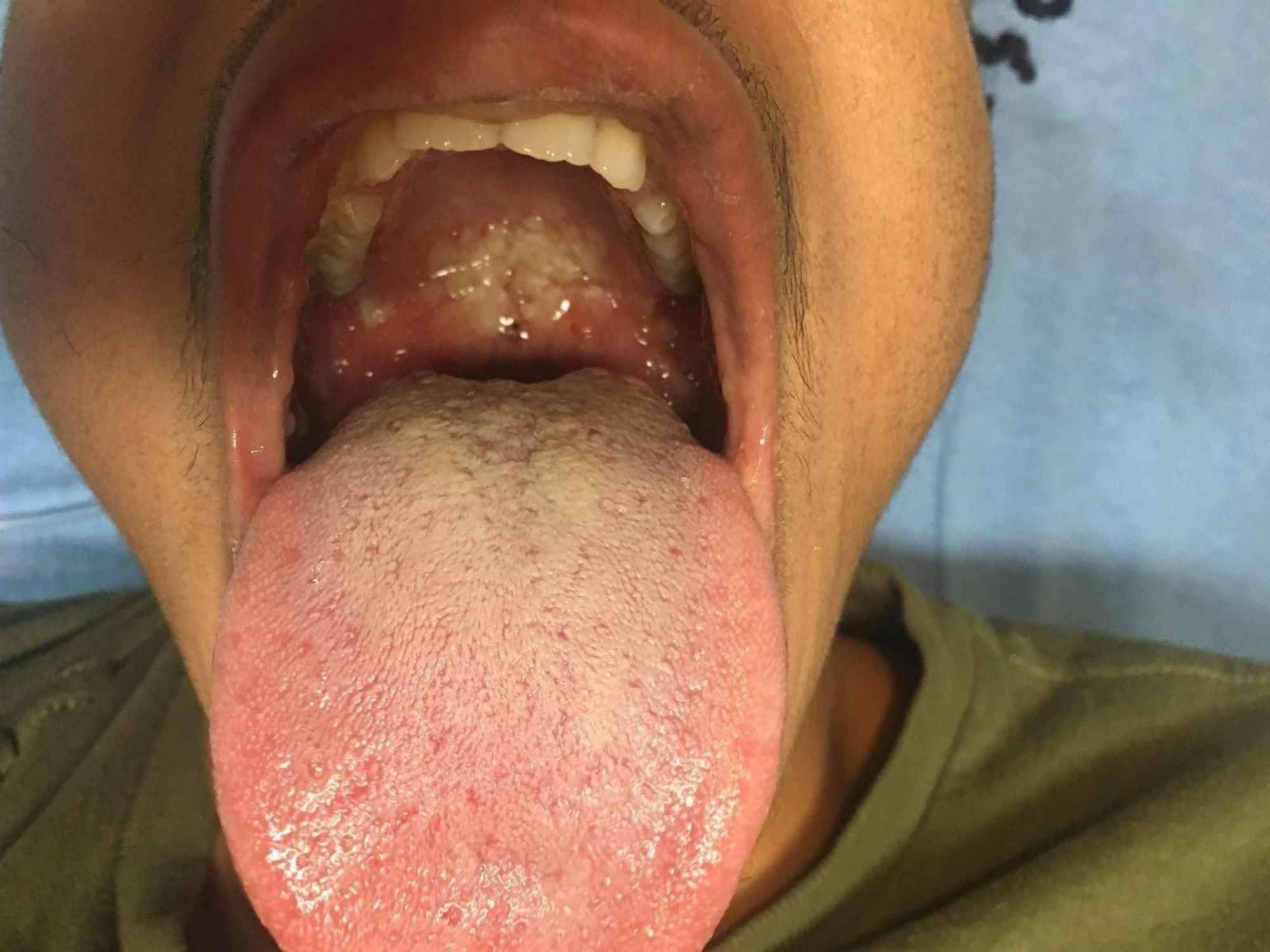
Oral cavity lesions

**Figure 3:**
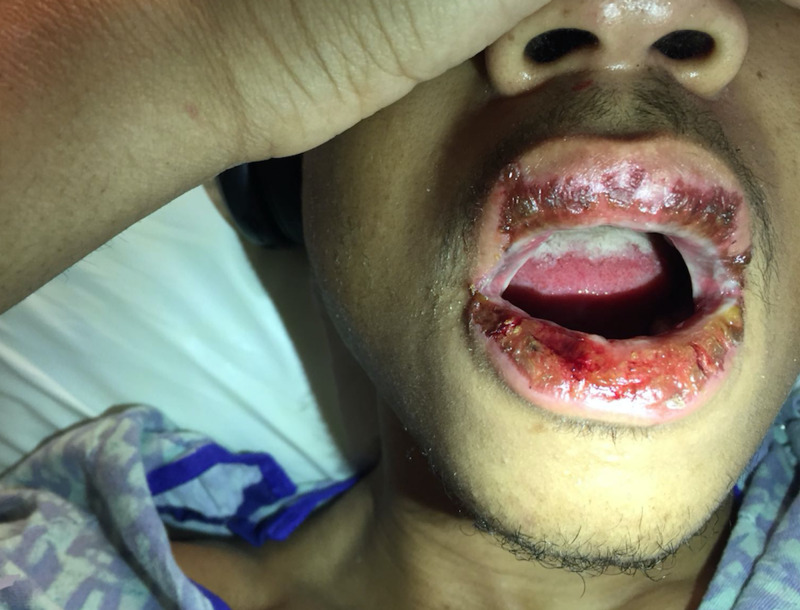
Oral rash and inflammation of the lips

He then presented to an urgent care facility when he began to develop genital lesions. A history of sexually transmitted diseases was suspected due to the patient’s lack of barrier protection use. He was tested for Neisseria gonorrhea and was treated prophylactically with intramuscular ceftriaxone. After discharge from the urgent care, he developed oral lesions as seen in Figures 1-3 and worsening genital lesions which prompted his visit to the Emergency Department on the same night. 

**Figure 4:**
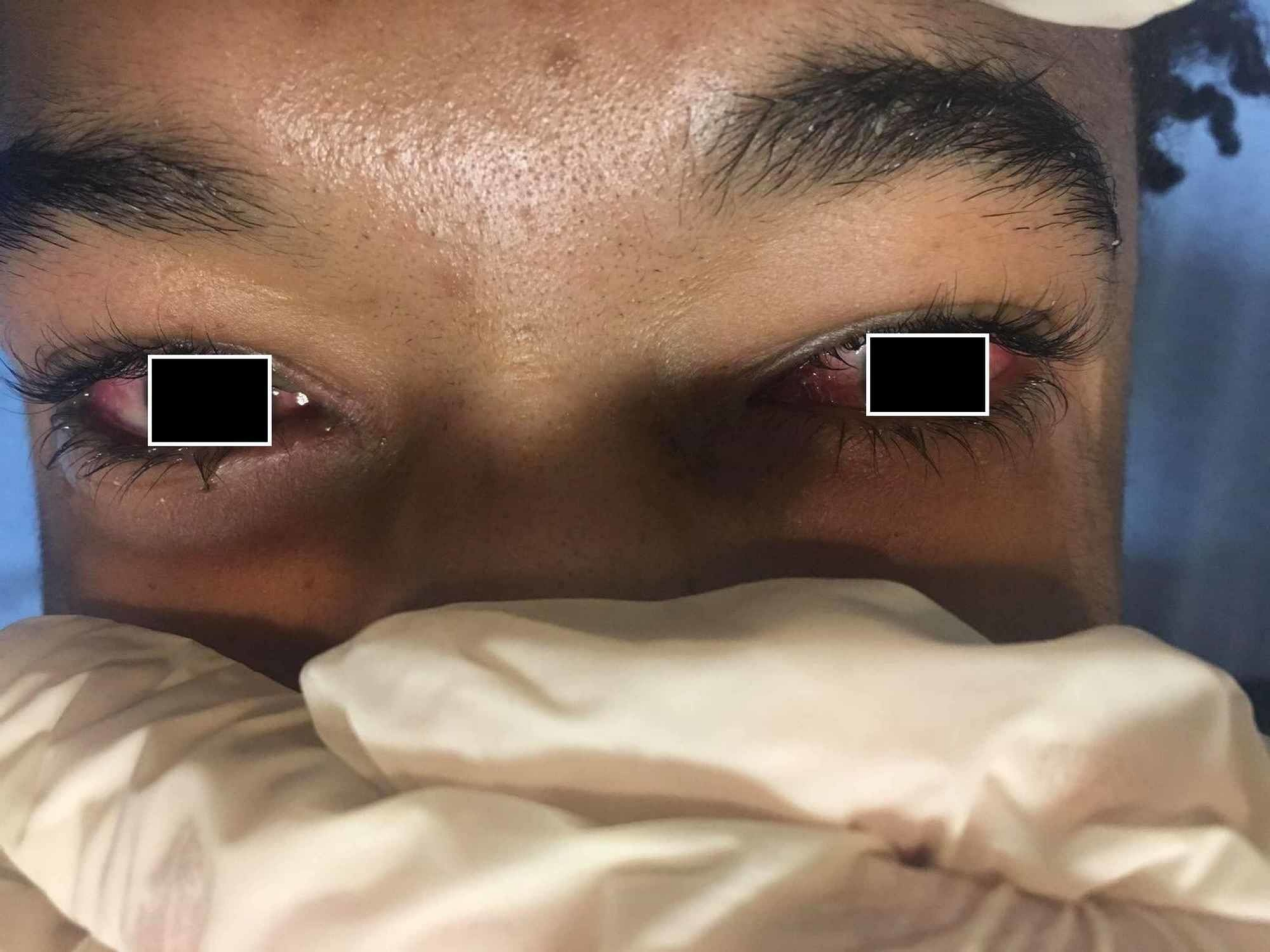
Bilateral conjunctivitis

The patient was tested for herpes simplex virus (HSV) (urethra), syphilis, chlamydia, gonorrhea, trichomonas, and Epstein-Barr virus (EBV) upon arriving in the Emergency Department. A rapid Strep test and throat culture was also performed. The results for all testing were negative. The patient was discharged home from the Emergency Department with amoxicillin and gentamicin eye drops. The following day, the patient returned back to the emergency room with worsening of all symptoms, which prompted inpatient admission to the hospital. Inpatient evaluation showed fever, weakness, fatigue, bilateral conjunctivitis (as seen in Figure 4), blurry vision, sore throat, nausea, dysphagia, drooling, dysuria, testicular swelling/pain, and ulcerated scrotal and penile lesions.

Routine laboratory workup revealed a leukocytosis of 17.41K/µL (normal: 4K/µL - 11K/µL) with neutrophilic predominance of 82.7%. Lactic acid, C-reactive protein, and erythrocyte sedimentation rate values were 2.3 mmol/L (normal: 0.5 mmol/L - 2.2 mmol/L), 15.3 mg/L (normal: 1 mg/L - 10 mg/L), and 20 mm/h (normal: < 15 mm/h), respectively. Screening for chlamydia, N. gonorrhoeae, human immunodeficiency virus (HIV), antinuclear antibody (ANA), hepatitis, human leukocyte antigen B27 (HLA-B27), and pathergy test for Behcet's were all negative. Mycoplasma pneumoniae IgM and IgG, herpes simplex virus (HSV)-1 IgG and IgM, and HSV-2 IgG were all positive.

Ceftriaxone and azithromycin were administered on admission. Acyclovir was started empirically for oral lesions. Intravenous (IV) methylprednisolone 50 mg was administered daily. The patient completed three days of IV Rocephin (ceftriaxone), 1 g (total) of IV azithromycin, five days of IV acyclovir, and he was then transitioned from IV methylprednisolone to oral steroids. After five days of treatment, complete resolution of symptoms was achieved and he was discharged home.

## Discussion

A detailed history and physical can be a useful tool when trying to differentiate between MPAM, EM, SJS, and TEN. Most patients with MPAM will have prodromal symptoms indicative of a respiratory infection approximately one week before the rash [4]. SJS and TEN are considered different spectrums of the same disease process which can also be preceded with prodromal symptoms of an upper respiratory infection, but there will often be a history of exposure to a new medication [4]. Medications associated with this disease process are antibiotics (sulfonamides, beta-lactams), nonsteroidal anti-inflammatory drugs (NSAIDs), allopurinol, antiepileptics, and nevirapine. In comparison, EM may be suggested if the patient admits to a history of herpes simplex infection.

In addition to mucosal involvement, 47% of MPAM patients present with a non-mucosal, cutaneous rash [7]. MPAM cutaneous rashes are different from other mucositis disease processes in their distribution. They are located more in the acral regions (46%) than on the trunk (23%) [7]. There are three types of MPAM in which all of them demonstrate clinical symptoms of atypical pneumonia, laboratory evidence of M. pneumoniae infection (elevated M. pneumoniae IgM antibodies, positive cultures, or M. pneumoniae cold agglutinins), and mucosal rash [4]. The delineation between the three presentations is determined by (1) a non-mucosal rash (classic MPAM) or (2) absence of a non-mucosal rash (MPAM sine rash). Severe MPAM is the third iteration and these patients possess both (1) and (2) above, along with greater than two sites of cutaneous lesions and widespread non-mucosal blisters or flat atypical target lesions [4]. Differentiating possible etiologies for mucosal lesions is important due to the radical differences in treatments and prognosis. MPAM is treated with antibiotics which most often resolves the disease process and the prognosis is very good.

## Conclusions

Mycoplasma mucositis is a rare but very important extrapulmonary manifestation of M. pneumoniae infection. We have presented the case of a patient who developed oral, ophthalmic, and genital lesions as a consequence of the M. pneumoniae infection. This pathology has been previously described as an atypical Stevens-Johnson syndrome without skin lesions. However, the condition is increasingly recognized as a separate entity, now termed Mycoplasma pneumoniae-associated mucositis (MPAM). Understanding the nuances of Mycoplasma mucositis is of significant importance to deliver the right treatment regimen to the patient and avoid costly and unnecessary testing. The clinical presentation of such a pathology is largely unknown in the medical community with few cases published. The authors hope that this report will lead to earlier identification of MPAM and its manifestations while adding useful clinical information to a growing body of literature on the subject.
